# Optical Coherence Tomography and Optical Coherence Tomography Angiography in Monitoring Coats' Disease

**DOI:** 10.1155/2017/7849243

**Published:** 2017-03-09

**Authors:** Wojciech Hautz, Joanna Gołębiewska, Beata Kocyła-Karczmarewicz

**Affiliations:** Department of Ophthalmology, The Children's Memorial Health Institute, Ul. Aleja Dzieci Polskich 20, Warsaw, Poland

## Abstract

*Purpose*. The aim of this study was to evaluate the usefulness of optical coherence tomography (OCT) and optical coherence tomography angiography (OCTA) in monitoring pediatric patients with Coats' disease. *Material and Methods*. This retrospective study included 9 Caucasian patients receiving treatment for Coats' disease at the Children's Memorial Health Institute Ophthalmology Department between December 2014 and May 2016. The course of the disease was monitored with OCTA in combination with OCT and fluorescein angiography (FA). *Results*. OCT B-scans obtained in all patients correlated with FA findings. Reliable OCTA images were obtained in 8 patients. In one patient, numerous artifacts due to poor visual acuity and retinal detachment confounded the interpretation of findings. *Conclusions*. OCTA and OCT, in combination with FA, are useful in Coats' disease diagnostics and treatment monitoring. As noninvasive methods, OCT and OCTA may be performed more often than FA, which enable precise monitoring of the disease and making decisions as to its further treatment.

## 1. Introduction

Coats' disease is a condition characterized by idiopathic congenital abnormalities in small retinal vessels followed by subretinal and intraretinal exudates. Untreated Coats' disease leads to loss of vision due to exudative retinal detachment, with 10% of patients developing secondary neovascular glaucoma [1]. Early diagnosis and treatment help preserve functional visual acuity [2–4]. Coats' disease is diagnosed mostly in children under 10 years of age. Over 75% of patients are males. Ninety-five percent of cases are unilateral. Initial anomalies found on fundus examination include areas of irregularly dilated small and medium retinal vessels. These idiopathic telangiectasias are typically located peripherally, in the temporal and lower quadrants, and affect the posterior pole in less than 5% of cases. In comparison with retinal areas affected by vascular anomalies, exudation is more extensive. Exudates tend to affect the macular area and contain cholesterol crystals, which are responsible for its yellow or yellowish-gray hue characteristic [5, 6].

The diagnosis, differential diagnoses, and treatment of Coats' disease are based not only on an ophthalmic examination, but also on imaging techniques: ultrasonography, fluorescein angiography (FA), and optical coherence tomography (OCT) [7]. Recently, ultrawide field angiography often replaces conventional FA to provide clinically useful information in Coats' disease [8].

Optical coherence tomography angiography (OCTA) is a new, noninvasive tool, based on split-spectrum amplitude-decorrelation angiography (SSADA), involving the detection and measurement of intravascular erythrocyte movement. OCTA delivers highly detailed, three-dimensional images of the entire microvasculature of the retina and choroid and helps assess retinal perfusion without intravenous dye injection [9–11]. This new technique is useful in the diagnosis of different retinal vascular diseases, such as diabetic retinopathy, retinal vein occlusion, or age-related macular degeneration [12–14]. OCTA seems to be a promising method also in the diagnostics of pediatric patients, for whom fluorescein angiography, as an invasive method, is particularly stressful [15]. The aim of the present study was to evaluate if OCTA can offer additional information about vascular disorders related to Coats' disease and to assess this method in diagnostics and monitoring children affected by Coats' disease.

## 2. Materials and Methods

This study was a retrospective analysis of 9 consecutive Caucasian patients (9 eyes) aged 8–17 (including 5 boys and 4 girls) treated for Coats' disease at our Ophthalmology Clinic between December 2014 and May 2016. The patients were divided into 2 groups. Group A included 4 children (cases 1–4) newly diagnosed with Coats' disease and never treated before. Group B included 5 children (cases 5–9) after treatment.

Every patient underwent a complete ophthalmological examination. The diagnosis of Coats' disease had been made based on the established criteria [16]. After fundus examination revealed the characteristic lesions, the patients underwent FA to confirm the findings, which is a necessary step for treatment initiation, and to determine more precise location of retinal telangiectasia. Cooperating patients had OCT and OCTA scans performed at the same time. The course of the disease had been monitored with OCTA in combination with OCT and FA. OCT and OCTA scans were also performed after every treatment cycle and at every follow-up visit, that is, every 3 months on average. If deemed necessary, FA was repeated during follow-up in some cases.

Fluorescein angiography was performed with TRC NW7 SF Retinal Camera (TOPCON) following intravenous injection of 10% sodium fluorescein solution, according to the established examination technique standards. Prior to dye administration, color fundus photographs were taken for each patient. The purpose of FA was to detect any central and peripheral retinal vessel anomalies, associated exudation, retinal avascular areas, and collateral circulation.

OCT and OCTA scans were performed using a commercially available RTVue XR Avanti with AngioVue (Optovue, Fremont, CA, USA), which captures 6 × 6 and 8 × 8 mm scanning area centred on the fovea. In some cases, 3 × 3 mm scans were created in regions of interest to improve visualization of flow anomalies.

In OCT scans, we assessed the extent and stage of central retinal lesions: the size of lipid deposits and the presence of intraretinal and subretinal fluid, hemorrhages, scars, retinovitreal proliferations, as well as areas of retinoschisis and retinal detachment.

In OCTA, superficial and deep retinal plexuses were assessed to detect the blood flow in central retinal vessels, the presence of anomalies in vessel size, vasodilatation, capillary dropout, and any hypoperfused areas. Manual adjustment of segmentation was also used to obtain clearer images. To define the characteristics of vascular abnormalities, two experienced readers reviewed the OCTA images independently. The findings of FA, OCT, and OCTA were correlated and compared for the importance of data they provided and their usefulness in diagnostics and treatment. Technical possibilities, limitations, and benefits of each method were also assessed.

All diagnostic examinations and treatment procedures were conducted in accordance with the established principles of good clinical practice after a written informed consent had been obtained from the patient's legal guardians.

## 3. Results

The characteristics of patients are summarized in Table 1.

**Figure 1:**
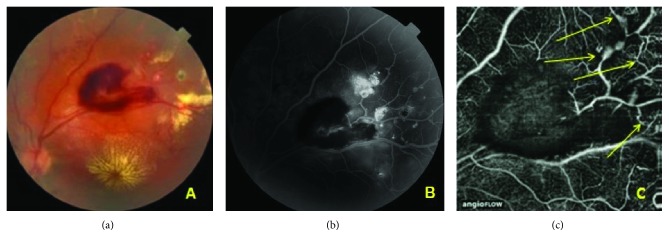
Case 1: central and peripheral lesions. (a) Color fundus photograph of the left eye. The upper temporal quadrant shows a large hemorrhage; lipid deposits in the macula and superior to the hemorrhage. (b) Late-phase FA. Multiple vascular anomalies in the upper temporal quadrant of the midperiphery of the retina. The vascular abnormalities located more peripherally are visible only in FA. (c) OCTA image of vessels in the upper temporal region. Superficial plexus. Vascular anomalies, dilated vessels and loops *(arrows)*. The retinal blood flow is partially obscured due to hemorrhage.

**Figure 2:**
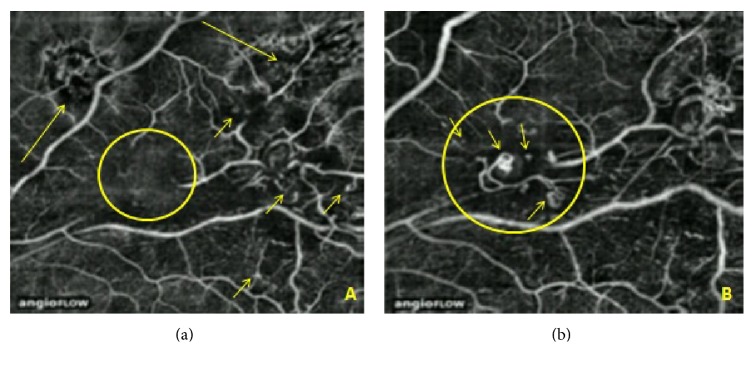
Case 1: serial OCTA analysis of the upper temporal region. Superficial plexus. (a) After initial treatment (1 TTT procedure). Retinal areas after TTT with faint flow *(long arrows)*. Hemorrhage, smaller than in Figure 1*(circle)*. Vascular dilatation and irregular blood flow can be seen temporal to the hemorrhage *(short arrows)*. (b) During treatment (after 3 TTT procedures). Additional vascular loops in the superficial plexus were visualized after hemorrhage resorption *(short arrows)*.

**Figure 3:**
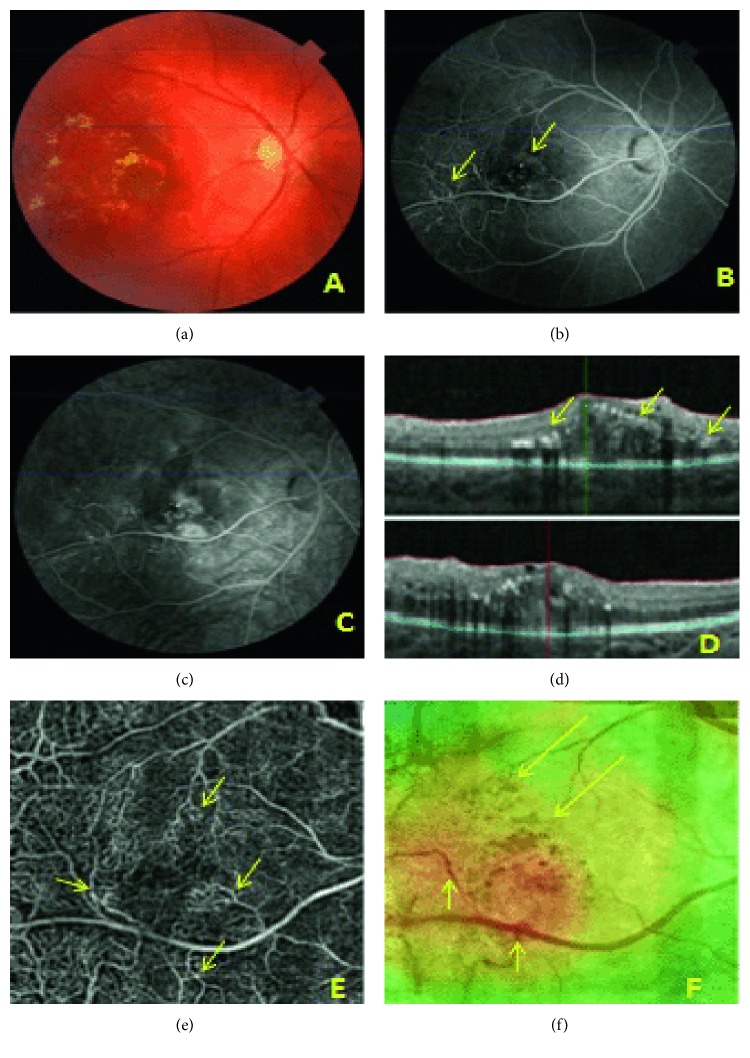
Case 2: prior to treatment, central retina. (a) Color fundus photograph of the right eye: macular edema and hard exudates. (b) Early-phase FA. Vascular anomalies near the macula and along temporal vessels *(arrows)*. (c) Late-phase FA. Leakage increasing over time. (d) OCT. Intraretinal fluid increasing retinal thickness and lipid deposits *(arrows).* (e) OCTA of the macular area. Superficial plexus: irregular vascular network with aneurysm-like dilations and vascular loops *(arrows)*; connections between the superior and inferior vascular branches. (f) Retinal thickness map superimposed on an en face image, increased retinal thickness *(red area)*, lipid exudates *(long arrows)*, and vascular anomalies *(short arrows)*.

**Figure 4:**
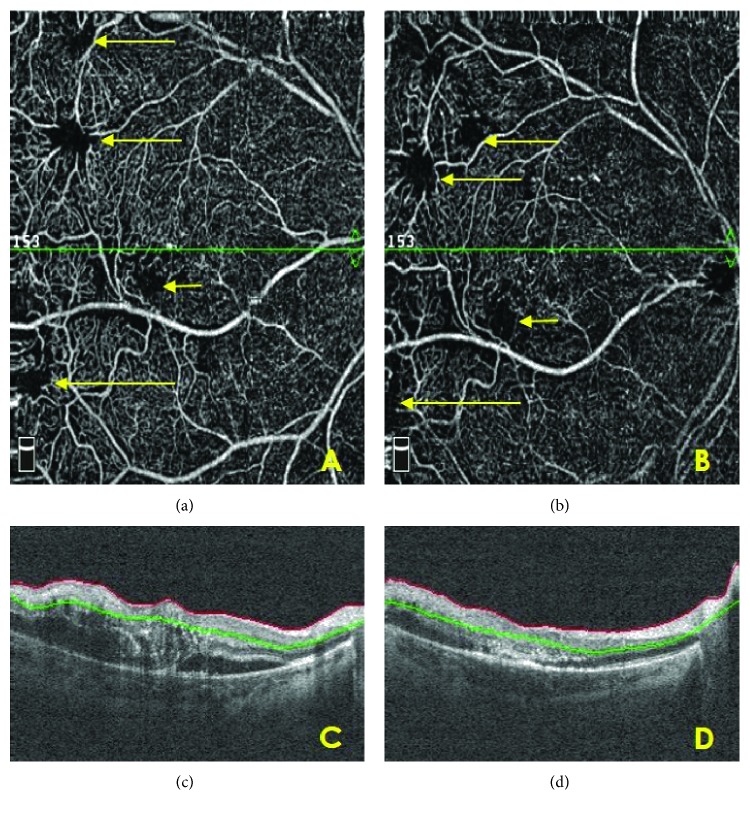
Case 2: posttreatment follow-up. (a), (b) Serial OCTA analysis of the macular region. Superficial plexus. After initial treatment (a) and after 3 TTT procedures (b). Retinal areas after TTT with faint flow—*long arrows*. Area of capillary hypoperfusion—*short arrow*. (c) OCT. The intraretinal and subretinal fluid with retinal thickening after initial treatment. (d) OCT. The decrease of retinal thickness and subretinal fluid regression after 3 TTT procedures.

Three children (cases 1, 2, and 8) had both central and peripheral retinal vessel anomalies (Figures 1–4).

All retinal pathologies found in affected eyes using FA, OCT, and OCTA techniques are summarized in Table 2.

OCTA and FA were comparable in terms of visualizing medium and large vascular abnormalities in the posterior pole. However, very small telangiectasias were not visible in OCTA, with FA proving to be a more accurate tool in their detection. Images of peripheral retinal lesions, located beyond the 8 × 8 mm OCTA scan area, were obtained in 2 very well cooperating patients (case 1 and case 2). Lesions located at the far periphery of the retina were not visible on OCTA images.

Vascular lesions limited exclusively to the peripheral retina were detected in six patients, with secondary pathologies observed in the macula (in group A, cases 3 and 4; in group B, cases 5, 6, 7, and 9). The differences in the ability of detecting vascular abnormalities in Coats' disease between FA and OCTA are summarized in Table 3.

Fundus pathologies observed in group A were vascular anomalies, lipid deposits, and serous macular detachment detected in all patients: hemorrhage in 1 patient (case 1; Figure 1) and partial peripheral retinal detachment in 2 patients (cases 3 and 4).

Group B findings included macular pathologies such as fibrotic scars (cases 5, 6, 7, and 9), intrascar vessels (cases 5 and 7), and areas of tractional retinal detachment (cases 7 and 9).

The treatment methods are summarized in Table 1.

Macular thickening, subretinal fluid, and exudates regressed during therapy. Serial analysis of OCT scans was useful during the follow-up (Figures 2 and 4). OCTA scans of superficial and deep retinal plexus following TTT showed the presence of faint perfusion in the retinal areas after TTT.

## 4. Discussion

Used since the 1960s, fluorescein angiography is the “gold standard” in choroidal and retinal vasculature assessment [17]. One definitive advantage of FA is the possibility of visualization even slight vascular pathologies of the posterior pole and retinal periphery. Nowadays, wide field angiography provides more detailed information on periphery retina in different vascular diseases, such as diabetic retinopathy or Coats' disease, and can replace conventional methods [8, 18]. One limitation of this technique is the relatively long duration of examination and the necessity of intravenous dye injection. Used since the 1990s, OCT, or a noninvasive “optical biopsy,” is an imaging technique involving optical scanning that facilitates a detailed assessment of the layered structure of the retina [19, 20]. The use of OCT in Coats' disease helps detect macular thickening and retinal detachment as well as locate lipid deposits [21]. It is an easy, short, and very useful technique that can be used for frequent follow-up assessments, especially in those children who tolerate the examination well. The key elements to assess macular pathology are evident on OCT; however, OCT B-scans alone do not help assess retinal microvasculature. The split-spectrum amplitude-decorrelation angiography (SSADA) algorithm in OCT scanning and the en face OCT technique, which helps visualize frontal cross-sections of the retina at various layers, contributed to the development of the noninvasive optical coherence tomography angiography for visualizing blood flow in retinal and choroidal vessels [22, 23]. Instruments for using OCT angiography in daily clinical practice have recently become available, and OCTA will probably revolutionise the diagnosis of retinal and choroidal diseases and replace invasive techniques in the future. To date, there are a lot of studies focusing on OCTA in retinal abnormalities in adults [24–26]. Lumbroso et al. described OCTA features in different macular pathologies, including Coats' disease [27]. Recently, Yonekawa et al. reported OCTA findings in an adult man suffering from Coats' disease [28]. Muakkassa et al. found the inner retinal vessels traversing an abnormal foveal avascular zone in four patients with unilateral Coats' disease, also in 2 normal fellow eyes [29]. Our study does not confirm the findings. To the best of our knowledge, only Stanga et al. described OCT angiography in typically pediatric diseases, such as X-linked retinoschisis, Best disease, and Coats' disease [15]. As far as we know, the present study is the first which investigates usefulness of both OCT and OCTA in monitoring Coats' disease in children.

Indisputable advantage of OCTA is a short duration of the examination—it takes only few seconds and, most of all, no need for the use of intravenous dye. This is particularly important in pediatric patients who have a poor tolerance of injections and find FA so stressful that sometimes the examination has to be stopped.

Vascular pathologies in Coats' disease involve both the superficial and deep retinal plexuses. OCTA scans allow detailed assessment of both of these plexuses, whereas FA fails to visualize the deep vascular plexus. OCTA images should always be assessed with OCT B-scans and en face OCT; only it guarantees precise evaluation of vascular pathologies. Although OCTA does not show leakage, macular oedema can be diagnosed together with OCT B-scans and en face OCT assessment. In OCTA faint flow in abnormal vessels after treatment, TTT scars were noticed but without subretinal and intraretinal fluid seen on OCT B-scans. So we concluded that the perfusion in the vessels is preserved, but without leaking. The main limitation of OCTA is that the technique affords no direct assessment of the vascular wall; instead, only the blood flow within vessels is visible. The use of central scans—with the maximum area of 8 × 8 mm—yields only images of the central retina. Moreover, poor visual acuity and poor fixation, which are common in Coats' disease, produce artifacts, which confound the interpretation of findings. Angiograms may not visualize vascular atrophy and vascular occlusion, where capillary or choroid capillary perfusion falls below the sensitivity threshold. In our group of patients, OCTA did not reveal any additional information regarding vascular abnormalities, and we do not notice any predictive value of OCTA in Coats' disease at that moment. Further observation is needed.

FA is a dynamic examination—due to the flow of the dye, the image of vessels changes dynamically. The examination shows vascular wall lesions, dye leakage points, areas of dye pooling, or staining, and, in early phases of FA, it affords a precise assessment of neovascularization. FA allows assessment of both the central and peripheral retina and affords interpretable images regardless of visual fixation problems.

A limitation of the study is a small sample of patients, but Coats' disease is a rare macular pathology. Additionally, it is difficult to obtain good quality OCT and OCTA images in very young patients. Because there are only few other reports describing OCTA features in Coats' disease, including smaller number of patients, mainly adults, it is difficult to compare our observation with those of the other authors. Further research is needed to describe more OCTA findings in Coats' disease.

## 5. Conclusions

OCTA in combination with FA and OCT can be useful in diagnosing and monitoring Coats' disease. OCTA results were in good agreement with the results of the FA, but OCT angiography failed to be a valid substitute for conventional angiography as a sole diagnostic method (the “gold standard”). The use of multimodal imaging, including these three techniques and color photography of the fundus, affords a comprehensive picture of Coats' disease pathologies.

## Figures and Tables

**Table 1:** Characteristics of patients.

Group	Case number	Age/years	Sex	Primary pathological changes		Diagnostic methods			Therapy
				Peripheral	Central	AF	OCT	OCTA	
A	1	16	F	VA LD RE SF H	VA LD RE	+	+	+	TTT
	2	8	M	VA LD RE SF	VA LD RE	+	+	+	TTT
	3	17	F	VA LD RE SF	LD RE SF	+	+	Poor visual acuity disables OCTA	TTT
	4	8	M	VA LD SF RE	LD	+	+	+	TTT Cryotherapy

B	5	11	M	VA LD	LD	+	+	+	TTT Cryotherapy
	6	12	F	VA LD SF RE	LD RS	+	+	+	TTT Cryotherapy
	7	14	M	VA LD RE SF	RE RS EM SF	+	+	+	TTT Cryotherapy Barrage laser treatment
	8	12	M	VA LD RE	VA LD RS EM	+	+	Poor visual acuity artifacts	TTT
	9	11	F	VA RE	RS EM	+	+	+	TTT Barrage laser treatment

F: female; M: male; VA: vascular anomalies; LD: lipid deposits; RE: retinal edema; H: hemorrhage; SF: subretinal fluid; RS: retinal scare; EM: epiretinal membrane.

**Table 2:** Detection of general pathologies using FA, OCT B-scans, and OCTA.

	FA	OCT	OCTA	Case number
Vascular anomalies	V	NV	V	1–9
Lipid deposits	Fluorescence blockage	V	V	1–6, 8
Epiretinal hemorrhage	Fluorescence blockage	V	Blockage of flow signal	1
Subretinal fluid	V	V	V	1–6, 7, 9
Retinal edema	V	V	V	1–9
Retinal scars	V	V	V	6–9
Epiretinal membrane	V	V	V	7–9

V: visible; NV: not visible.

**Table 3:** Ability of detecting vascular lesions, FA versus OCTA.

Diagnostic method	Far periphery	Posterior pole, scan 8 × 8, 6 × 6 mm		
		Very small VA	Medium size VA	Large VA
FA	Visible	Visible	Visible	Visible
OCTA	Not visible	Not visible	Visible	Visible

VA: vascular abnormalities.
